# fMRI-compatible rehabilitation hand device

**DOI:** 10.1186/1743-0003-3-24

**Published:** 2006-10-06

**Authors:** Azadeh Khanicheh, Andrew Muto, Christina Triantafyllou, Brian Weinberg, Loukas Astrakas, Aria Tzika, Constantinos Mavroidis

**Affiliations:** 1 Department of Mechanical and Industrial Engineering, Northeastern University, Boston, MA, USA; 2 Athinoula A. Martinos Center for Biomedical Imaging, Harvard Medical School, Boston, MA, USA; 3 NMR Surgical Laboratory Massachusetts General Hospital, Harvard Medical School, Boston, MA, USA

## Abstract

**Background:**

Functional magnetic resonance imaging (fMRI) has been widely used in studying human brain functions and neurorehabilitation. In order to develop complex and well-controlled fMRI paradigms, interfaces that can precisely control and measure output force and kinematics of the movements in human subjects are needed. Optimized state-of-the-art fMRI methods, combined with magnetic resonance (MR) compatible robotic devices for rehabilitation, can assist therapists to quantify, monitor, and improve physical rehabilitation. To achieve this goal, robotic or mechatronic devices with actuators and sensors need to be introduced into an MR environment. The common standard mechanical parts can not be used in MR environment and MR compatibility has been a tough hurdle for device developers.

**Methods:**

This paper presents the design, fabrication and preliminary testing of a novel, one degree of freedom, MR compatible, computer controlled, variable resistance hand device that may be used in brain MR imaging during hand grip rehabilitation. We named the device MR_CHIROD (**M**agnetic **R**esonance **C**ompatible Smart **H**and **I**nterfaced **R**ehabilitation **D**evice). A novel feature of the device is the use of Electro-Rheological Fluids (ERFs) to achieve tunable and controllable resistive force generation. ERFs are fluids that experience dramatic changes in rheological properties, such as viscosity or yield stress, in the presence of an electric field. The device consists of four major subsystems: a) an ERF based resistive element; b) a gearbox; c) two handles and d) two sensors, one optical encoder and one force sensor, to measure the patient induced motion and force. The smart hand device is designed to resist up to 50% of the maximum level of gripping force of a human hand and be controlled in real time.

**Results:**

Laboratory tests of the device indicate that it was able to meet its design objective to resist up to approximately 50% of the maximum handgrip force. The detailed compatibility tests demonstrated that there is neither an effect from the MR environment on the ERF properties and performance of the sensors, nor significant degradation on MR images by the introduction of the MR_CHIROD in the MR scanner.

**Conclusion:**

The MR compatible hand device was built to aid in the study of brain function during generation of controllable and tunable force during handgrip exercising. The device was shown to be MR compatible. To the best of our knowledge, this is the first system that utilizes ERF in MR environment.

## Background

Functional magnetic resonance imaging (fMRI) has been widely used to investigate human brain mechanisms controlling voluntary movement in humans, and reorganization of this system in response to neurological injury, such as stroke and Parkinson's disease [1]. Study of motor performance in controllable dynamic environments during fMRI could provide important insights into human motor control and assist in the development of optimal rehabilitation devices and exercise protocols. This motivates the development of robotic/mechatronic interfaces, which can control and measure force during movements in humans and quantify the kinematics of motor task performance while performing fMRI [2]. However, the engineering challenges involved with these procedures are that the robotic and mechatronic devices need to be "compatible" with the MR environment.

Conventional rehabilitation robotic and mechatronic devices are typically not feasible for use in an MR environment because they introduce electromagnetic interfaces, in three primary forms [3]. Any robotic device within the MR environment is exposed to a strong static magnetic field of 1.5 to 3 T and substantial forces will be sensed by a device that contains any ferromagnetic component, potentially introducing a safety hazard. Secondly, conventional materials, actuators, and sensors have the potential to emit radio frequency energy that can be easily detected by an MRI scanner and will disturb the image quality and result in significant image artifacts. Third, strong magnetic fields can affect the successful operation of the robotic device and result in poor performance. Therefore, from an engineering point of view the development of MR compatible robotic and mechatronic devices is not trivial at all as each system component needs to be selected appropriately and tested for MR compatibility. Of special difficulty is the MR compatibility of sensors and actuators that not only have to be made out of MR compatible materials but their principle of operation should not affect or be affected by the MR environment as well.

Several examples of MR compatible robotic devices have been demonstrated for surgical applications [4-8]. A non-portable, haptic interface compatible with fMRI that uses a hydraulic master-slave system to power the robot remotely, from the outside of a MR room, was presented in [9,10]. An fMRI compatible virtual reality system that included a data glove equipped with tactile feedback was developed in [11,12]. The glove was able to collect data from the patient's hand motions and transfer information through tactile feedback. However, it was not able to apply forces and torques required in exercises of motor rehabilitation. An MR compatible stationary bicycle for exercising inside an open magnet was presented in [13]. Even though the device was equipped with sensors that could obtain data during the patient's exercise and even if it could provide variable resistance through purely mechanical means (variable – pre-adjusted friction), it was clearly a non-portable device, that can only operate in open magnets and had no computer controlled torque generation for real time variable torque exercises. A robotic arm compatible with fMRI that uses two-way, air-driven cylinders, servo valves, and linkages has been presented to study brain regions involved in processing reach errors [14]. The force on the handle of the robot is controlled by the inputs of the servo valves. A haptic interface device for fMRI studies has been presented in [15]. The device uses two coils that produce a Lorentz force induced by the large static magnetic field of the MR scanner. Devices utilizing this type of force actuation are very sensitive to their placement and orientation within the MRI scanner's magnetic field, which significantly limits the range of motion. Also, several fMRI compatible force sensing systems have been developed to measure forces exerted by subjects in their upper extremities for motor function studies [16,17]. These systems use sensors to quantify forces and they don't utilize any actuators to apply forces.

## Methods

The current study aimed to develop a portable, computer controlled, variable resistance, MR compatible hand device to evaluate activation in motor cortex regions during handgrip rehabilitation. We named the device MR_CHIROD (Magnetic Resonance Compatible Smart Hand Interfaced Rehabilitation Device). This paper focuses on the design, fabrication, and preliminary MR compatibility testing of MR_CHIROD. A key feature of the device is the use of electro rheological fluids (ERF) to achieve computer controlled, resistive force generation. Here, we demonstrate for the first time that ERFs are fully MR compatible. Our study, also, demonstrates that there is neither an effect from the MR environment on the performance of the MR_CHIROD (including its position and force sensors), nor significant degradation on MR images by the introduction of the MR_CHIROD in the MR scanner. Tests with the first prototype of MR_CHIROD showed that it was able to provide 160 N resistive forces, which is approximately 50% of the maximum level of gripping force that a human hand can apply.

### Electro Rherological Fluids

Electro-rheological fluids (ERFs) are fluids that experience dramatic changes in rheological properties, such as viscosity, in the presence of an electric field. Willis M. Winslow first explained the effect in the 1940's using oil dispersions of fine powders [18]. The fluids are made from suspensions of an insulating base fluid and particles on the order of one tenth to one hundred microns (in size). In the presence of an electric field, the particles, due to an induced dipole moment, rearrange into a more organized manner, or form chains along the field lines. These chains alter the ERF's viscosity, yield stress, and other properties, allowing the ERF to change consistency from that of a liquid to a viscoelastic gel, with response to changes in electric fields on the order of milliseconds.

Under zero field conditions an ERF is generally characterized by a simple Newtonian viscosity. When subjected to high electric fields, the ERF alters its state from Newtonian oil to a non-Newtonian Bingham plastic. As a Bingham plastic, the ERF exhibits a linear relationship between stress and strain rate like a Newtonian fluid, but only after a minimum required yield stress is exceeded. Before that point, it behaves as a solid. This shear stress behavior of an electro-rheological fluid is described most simply by the well known Bingham Model:

*τ *= *τ*_*y *_+ *η*γ˙
 MathType@MTEF@5@5@+=feaafiart1ev1aaatCvAUfKttLearuWrP9MDH5MBPbIqV92AaeXatLxBI9gBaebbnrfifHhDYfgasaacH8akY=wiFfYdH8Gipec8Eeeu0xXdbba9frFj0=OqFfea0dXdd9vqai=hGuQ8kuc9pgc9s8qqaq=dirpe0xb9q8qiLsFr0=vr0=vr0dc8meaabaqaciaacaGaaeqabaqabeGadaaakeaaiiGacuWFZoWzgaGaaaaa@2E63@     (1)

where, *τ *is shear stress; *τ*_*y *_is the yield stress, *η *is the dynamic viscosity and, γ˙
 MathType@MTEF@5@5@+=feaafiart1ev1aaatCvAUfKttLearuWrP9MDH5MBPbIqV92AaeXatLxBI9gBaebbnrfifHhDYfgasaacH8akY=wiFfYdH8Gipec8Eeeu0xXdbba9frFj0=OqFfea0dXdd9vqai=hGuQ8kuc9pgc9s8qqaq=dirpe0xb9q8qiLsFr0=vr0=vr0dc8meaabaqaciaacaGaaeqabaqabeGadaaakeaaiiGacuWFZoWzgaGaaaaa@2E63@, is the shear rate.

The yield stress, *τ*_*y*_, and the dynamic viscosity, *η*, are two of the most important parameters that effect the design of ERF based devices. The dynamic viscosity, *η*, is mostly determined by the base fluid with some field dependency, which is neglected when using the Bingham Model. The field-induced yield stress, *τ*_*y*_, depends on the field strength and is generally considered shear rate independent.

During the last ten years, some researchers proposed the use of ERFs in an effort to improve the performance of haptic, force-feedback and rehabilitation devices [19-24]. Our team has developed several concepts and prototypes of ERF based haptic systems including a haptic knob and a two degree of freedom joystick [25-28]. The use of ERF actuating/resistive elements and brakes in rehabilitation has been very limited. The majority of the rehabilitations devices employing ERF elements that have been developed so far were fixed based, non-portable, non-wearable systems [29-31]. Our team developed the first wearable ERF driven knee rehabilitation device called AKROD (Active Knee Rehabilitation Orthotic Device) [32].

### Hand device design

The proposed design for the novel hand rehabilitation device that is called MR_CHIROD consists of four major subsystems: a) an ERF resistive element; b) a gearbox; c) two handles and d) two sensors, one optical encoder and one force sensor, to measure the patient induced motion and force. Each subsystem includes several components of varying complexity. In general, all of the components were designed, with strength and safety in mind, to be MR compatible, and optimized for regular and high-stress testing.

**Figure 1:**
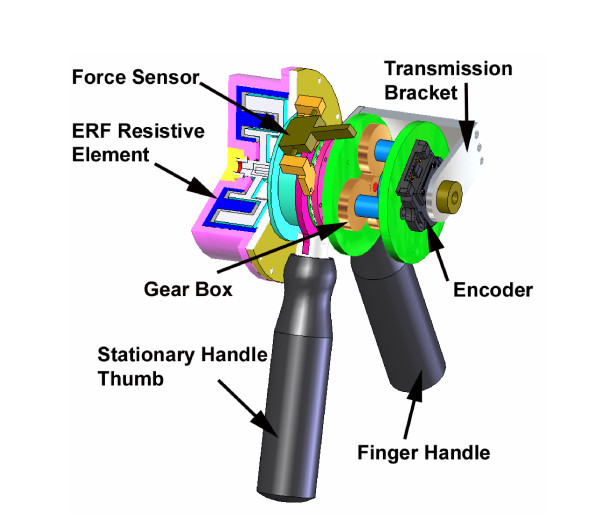
MR compatible ERF driven hand device.

The maximum gripping force that can be generated by the hand in males is 400 N and in females 228 N [33]. All components were designed so that the device is capable of applying 150 N of force at the human operator's hand holding the device's handles (approximately 50% of a healthy hand's gripping force). All CAD models and mechanism analysis were performed using Solid Works. The complete CAD model for the MR_CHIROD is shown in Fig. 1. A detailed description of the device components and the system characteristics of the MR_CHIROD are summarized in Table 1.

**Table 1:** System Characteristics of MR_CHIROD

**ERF Resistive Element Parameters:**	
Number of concentric cylinders	2
Gap between cylinders	1.25 [mm]
Outer diameter of resistive element	37.24 [mm]
Height of resistive element	21 [mm]
Max. resistive element torque (at 2 kV)	0.4 [N ml

**Overall Device Parameters**	

Gear ratio	31.6:1
Handle length	0.08 [m]
Max. device resistive force	160 [N]

#### ERF resistive element design

**Figure 2:**
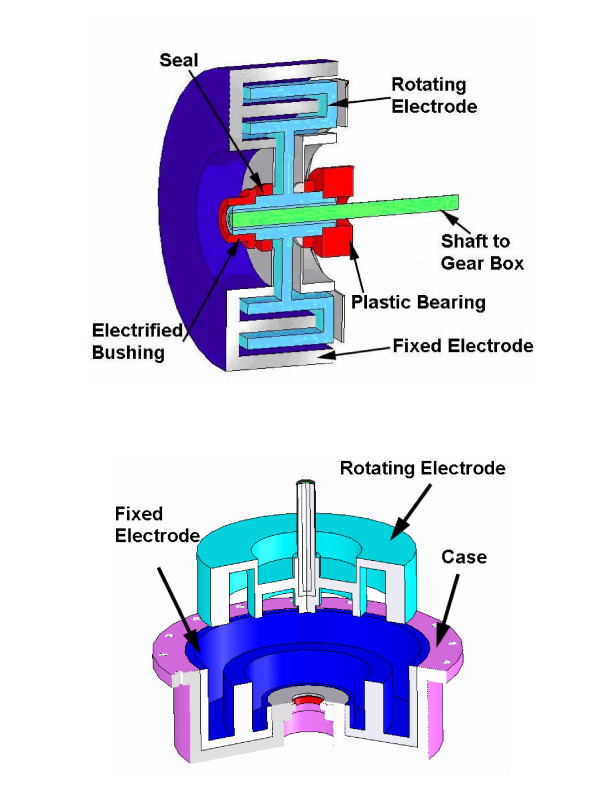
**CAD drawing of the rotary resistive element**. without its nylon case and all its components assembled (top); with its nylon case placed around fixed electrode and the rotating electrode just before being inserted in the fixed electrode (bottom).

The unique controllable variable resistance of MR_CHIROD is achieved through an ERF element that connects to the output of the gear system. Using the electrically controlled rheological properties of ERFs, compact resistive elements capable of supplying high resistive and controllable torques, were developed. The MR_CHIROD uses a rotary ERF resistive element to control the resistive torque. The resistive element consists of stationary and fixed aluminum electrodes, which were configured in a concentric circular pattern (Fig. 2). The development of the ERF resistive element was based on our previous work to develop such components for haptic interfaces used in the car industry and for smart knee orthoses [25-28,32]. However the proposed design for the rotary ERF resistive element that is used in this paper has novel features regarding the geometry of the electrodes, due to the small size constraints involved with the hand rehabilitation.

In our previous work, we have used the simple concept of a concentric cylinder (CC) rotary resistive element consisting of two concentric cylinders acting as electrodes, one fixed and one rotating [27]. The inner and outer cylindrical electrodes were separated only by a thin layer of fluid and applying an electric field across the gap altered the fluid's properties. More specifically, the fluid's yield stress was increased. When the rotating cylinder was in motion, the higher yield stress corresponded to an increased shear stress on the electrode's surfaces, which eventually created increased resistance at the human operator hands. Shear forces are directly proportional to the surface area of the electrodes, so to maximize the torque/force output from the ERF-based resistive element, the surface area of the electrodes needs to be increased while the volume of the whole system is as small as possible.

In this project, we used a novel way to maximize the surface area of the electrodes while keeping the resistive element's overall size as small as possible. We employed the concept of two electrodes, each one being a set of multiple concentric cylinders. One of the electrodes served as the fixed one and was located on the external side of the resistive element, while the second electrode served as the rotating one that could "mate" with the fixed one so that several consecutive pairs of concentric cylinders are formed. Fig. 2 (bottom) shows each one of the electrodes, fixed and rotating, with each one's concentric cylinders ready to be "mated". The multiple concentric cylinder design for the two electrodes allows for maximum shearing surface area while maintaining a compact overall volume for the resistive element. The actuation of the viscous fluid occurs within the very small gap between consecutive cylinders of the fixed and rotating electrodes and is consequently creating a resistive torque on the rotating shaft. By manipulating the strength of the electric field applied on the fluid, at each pair of consecutive concentric cylinders, the torque can be easily controlled.

In order to estimate the dimensions of the ERF resistive element, a mathematical model was derived using the Bingham model.

*T *= *Fr*_*rot *_= [*τA*_*rot*_]*r*_*rot *_= *τ*_*y*_*A*_*rot *_*r*_*rot *_+ *η*γ˙
 MathType@MTEF@5@5@+=feaafiart1ev1aaatCvAUfKttLearuWrP9MDH5MBPbIqV92AaeXatLxBI9gBaebbnrfifHhDYfgasaacH8akY=wiFfYdH8Gipec8Eeeu0xXdbba9frFj0=OqFfea0dXdd9vqai=hGuQ8kuc9pgc9s8qqaq=dirpe0xb9q8qiLsFr0=vr0=vr0dc8meaabaqaciaacaGaaeqabaqabeGadaaakeaaiiGacuWFZoWzgaGaaaaa@2E63@*A*_*rot*_*r*_*rot *_    (2)

where, *T*, is the torque output, *F *is the force, *A*_*rot *_is the rotating surface area, and *η*, γ˙
 MathType@MTEF@5@5@+=feaafiart1ev1aaatCvAUfKttLearuWrP9MDH5MBPbIqV92AaeXatLxBI9gBaebbnrfifHhDYfgasaacH8akY=wiFfYdH8Gipec8Eeeu0xXdbba9frFj0=OqFfea0dXdd9vqai=hGuQ8kuc9pgc9s8qqaq=dirpe0xb9q8qiLsFr0=vr0=vr0dc8meaabaqaciaacaGaaeqabaqabeGadaaakeaaiiGacuWFZoWzgaGaaaaa@2E63@, *r*_*rot*_, *τ*, *τ*_*y *_are the dynamic viscosity, the shear rate, the radius of rotating electrode, the shear stress, and the yield stress, respectively. The area of the rotating surface electrode is: *A *= 2*πr*_*rot*_*l *where *l *is the length of the rotating electrode surface.

The shear rate, γ˙
 MathType@MTEF@5@5@+=feaafiart1ev1aaatCvAUfKttLearuWrP9MDH5MBPbIqV92AaeXatLxBI9gBaebbnrfifHhDYfgasaacH8akY=wiFfYdH8Gipec8Eeeu0xXdbba9frFj0=OqFfea0dXdd9vqai=hGuQ8kuc9pgc9s8qqaq=dirpe0xb9q8qiLsFr0=vr0=vr0dc8meaabaqaciaacaGaaeqabaqabeGadaaakeaaiiGacuWFZoWzgaGaaaaa@2E63@, defined as: ωrrotg
 MathType@MTEF@5@5@+=feaafiart1ev1aaatCvAUfKttLearuWrP9MDH5MBPbIqV92AaeXatLxBI9gBaebbnrfifHhDYfgasaacH8akY=wiFfYdH8Gipec8Eeeu0xXdbba9frFj0=OqFfea0dXdd9vqai=hGuQ8kuc9pgc9s8qqaq=dirpe0xb9q8qiLsFr0=vr0=vr0dc8meaabaqaciaacaGaaeqabaqabeGadaaakeaadaWcaaqaaGGaciab=L8a3jabdkhaYnaaBaaaleaacqWGYbGCcqWGVbWBcqWG0baDaeqaaaGcbaGaem4zaCgaaaaa@35CF@ where, *ω *is the angular velocity, and *g *is the gap width between the rotating and the fixed electrodes.

The torque output equation of the concentric cylinder rotary resistive element using these variables is:

T=2lπrrot2[τy+η(ωrrotg)]     (3)
 MathType@MTEF@5@5@+=feaafiart1ev1aaatCvAUfKttLearuWrP9MDH5MBPbIqV92AaeXatLxBI9gBaebbnrfifHhDYfgasaacH8akY=wiFfYdH8Gipec8Eeeu0xXdbba9frFj0=OqFfea0dXdd9vqai=hGuQ8kuc9pgc9s8qqaq=dirpe0xb9q8qiLsFr0=vr0=vr0dc8meaabaqaciaacaGaaeqabaqabeGadaaakeaacqWGubavcqGH9aqpcqaIYaGmcqWGSbaBiiGacqWFapaCcqWGYbGCdaqhaaWcbaGaemOCaiNaem4Ba8MaemiDaqhabaGaeGOmaidaaOWaamWaceaacqWFepaDdaWgaaWcbaGaemyEaKhabeaakiabgUcaRiab=D7aOnaabmGabaWaaSaaaeaacqWFjpWDcqWGYbGCdaWgaaWcbaGaemOCaiNaem4Ba8MaemiDaqhabeaaaOqaaiabdEgaNbaaaiaawIcacaGLPaaaaiaawUfacaGLDbaacaWLjaGaaCzcamaabmGabaGaeG4mamdacaGLOaGaayzkaaaaaa@5028@

Every type of ERF is composed of a different composition of suspended particles in a fluid base and thus has its own unique behaviour and properties. Therefore, each ERF has its own yield strength and dynamic viscosity characteristic relationships. After testing the fluid used in this project and determining its properties, the final modelling equation for the concentric cylinder resistive element using this fluid is:

T=2lπrrot2[(0.044E2+0.3378E+τf)+η(ωrrotg)]     (4)
 MathType@MTEF@5@5@+=feaafiart1ev1aaatCvAUfKttLearuWrP9MDH5MBPbIqV92AaeXatLxBI9gBaebbnrfifHhDYfgasaacH8akY=wiFfYdH8Gipec8Eeeu0xXdbba9frFj0=OqFfea0dXdd9vqai=hGuQ8kuc9pgc9s8qqaq=dirpe0xb9q8qiLsFr0=vr0=vr0dc8meaabaqaciaacaGaaeqabaqabeGadaaakeaacqWGubavcqGH9aqpcqaIYaGmcqWGSbaBiiGacqWFapaCcqWGYbGCdaqhaaWcbaGaemOCaiNaem4Ba8MaemiDaqhabaGaeGOmaidaaOWaamWaceaacqGGOaakcqaIWaamcqGGUaGlcqaIWaamcqaI0aancqaI0aancqWGfbqrdaahaaWcbeqaaiabikdaYaaakiabgUcaRiabicdaWiabc6caUiabiodaZiabiodaZiabiEda3iabiIda4iabdweafjabgUcaRiab=r8a0naaBaaaleaacqWGMbGzaeqaaOGaeiykaKIaey4kaSIae83TdG2aaeWaceaadaWcaaqaaiab=L8a3jabdkhaYnaaBaaaleaacqWGYbGCcqWGVbWBcqWG0baDaeqaaaGcbaGaem4zaCgaaaGaayjkaiaawMcaaaGaay5waiaaw2faaiaaxMaacaWLjaWaaeWaceaacqaI0aanaiaawIcacaGLPaaaaaa@6129@

where, *τ*_*f *_is the no-field frictional yield stress, *η *is the dynamic viscosity of the fluid and is equal to 167 [cp] for the chosen ERF, and *E*, is the electric field, governed by the relationship:

E=Vrrotln(rori)[kV/mm]     (5)
 MathType@MTEF@5@5@+=feaafiart1ev1aaatCvAUfKttLearuWrP9MDH5MBPbIqV92AaeXatLxBI9gBaebbnrfifHhDYfgasaacH8akY=wiFfYdH8Gipec8Eeeu0xXdbba9frFj0=OqFfea0dXdd9vqai=hGuQ8kuc9pgc9s8qqaq=dirpe0xb9q8qiLsFr0=vr0=vr0dc8meaabaqaciaacaGaaeqabaqabeGadaaakeaafaqabeqacaaabaGaemyrauKaeyypa0ZaaSaaaeaacqWGwbGvaeaacqWGYbGCdaWgaaWcbaGaemOCaiNaem4Ba8MaemiDaqhabeaaieGakiab=XgaSjab=5gaUjabcIcaOmaalaaabaGaemOCai3aaSbaaSqaaiabd+gaVbqabaaakeaacqWGYbGCdaWgaaWcbaGaemyAaKgabeaaaaGccqGGPaqkaaaabaGaei4waSLaee4AaSMaeeOvayLaei4la8IaeeyBa0MaeeyBa0Maeiyxa0faaiaaxMaacaWLjaWaaeWaceaacqaI1aqnaiaawIcacaGLPaaaaaa@4D11@

where, *V*, is the voltage and, *r*_*i *_and *r*_*o *_are the radius of the inner and outer cylindrical electrodes.

The output torque of the ERF resistive element consisting of multiple concentric cylinders is calculated from the summation of the torques obtained from each pair of rotating and fixed electrodes:

T=∑i=1NTi     (6)
 MathType@MTEF@5@5@+=feaafiart1ev1aaatCvAUfKttLearuWrP9MDH5MBPbIqV92AaeXatLxBI9gBaebbnrfifHhDYfgasaacH8akY=wiFfYdH8Gipec8Eeeu0xXdbba9frFj0=OqFfea0dXdd9vqai=hGuQ8kuc9pgc9s8qqaq=dirpe0xb9q8qiLsFr0=vr0=vr0dc8meaabaqaciaacaGaaeqabaqabeGadaaakeaacqWGubavcqGH9aqpdaaeWbqaaiabdsfaunaaBaaaleaacqWGPbqAaeqaaaqaaiabdMgaPjabg2da9iabigdaXaqaaiabd6eaobqdcqGHris5aOGaaCzcaiaaxMaadaqadiqaaiabiAda2aGaayjkaiaawMcaaaaa@3C1B@

where *T*_*i *_is the torque calculated from Equation (4) for one pair of fixed and rotating cylindrical surfaces, and *N *is the number of concentric cylinders of the rotating electrode.

As it is shown in the above, the output torque of the ERF resistive element is the function of an electric field (the input voltage sent to the electrodes), the geometry of the resistive element (radius and length of electrodes), shear rate (angular velocity of the rotating electrode), and the properties of the ERF itself. Approximately 50% of a healthy hand's gripping force (150 N) was selected as the design goal for the smart hand device. From knowledge gained with previous ERF resistive elements [26], the gap size was set to 1.25 mm and the maximum voltage to 2 kV. The average squeezing rate is 0.5 Hz, meaning an angular velocity, *ω*, of approximately 2.6 rad/s. With all those in place, the design of the ERF resistive element was simplified down to the selection of three variables: the number of concentric cylinders in the positive and the negative electrode, the radius of the electrodes, and the length of the electrodes. Considering the small size constraints and performing the parametric studies using the mathematical model, the dimensions of the ERF resistive element were selected as shown in Table 1.

#### Gear box design

A gearbox was used to increase the resistive torque dissipated by the system. The resistive torque coming from the ERF resistive element is relatively small compared to what can be generated from a human hand. A resistive torque element without a gearbox would need to be excessively large. To keep the volume and weight of the entire device small, a large ratio gear box, 1 to 31.6 was designed. The gear system multiplies the ERF resistive torque and also serves as the foundation for the sensor sub-systems.

#### Handle design

The handles are the haptic interface for the operator. They are designed to rotate 75 degrees about the center axis and were balanced at the center of mass. The thumb grip is the stationary grip and the center of mass of MR_CHIROD is well centered just above the column of the grip. The hand grip assembly allows one degree of freedom and transmits the largest torque of the system to the input of the gearbox. The transmission bracket rotates about the axis and transmits the forces from the handles to the gearbox. The handles are made of garolite, which stands the high forces and is MR compatible.

#### Sensors

**Figure 3:**
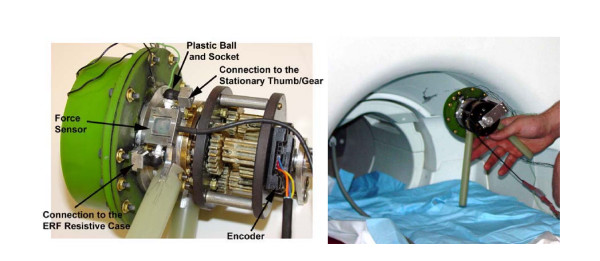
Prototype of the ERF driven MR_CHIROD.

All of the necessary clinical data can be obtained by employing two primary sensors into the device design. The first is an optical encoder (Fig. 3) to measure angles, velocities, and accelerations of the hand. The optical encoder is attached to the input side of the gearbox and gives a direct reading of the handles position. The ideal sensor, which has been included into the present design, is a Renco Low-Profile Encoder with a 1024 resolution.

The second sensor is a miniature force sensor for measuring the gripping strength of the patients' hand and for closed-loop control of the ERF resistive element. The FUTEK force sensor (aluminum strain gage) links the stationary thumb grip/gearbox to the ERF resistive element via two parallel surfaces either loading the sensor in tension or compression (Fig. 3). The force sensor is supported at two ends with 3 degree of freedom (rotation) ball and socket pin connections so that the gage is loaded as a two force member. The pin connections are attached to the hand device by plastic sockets so that the force sensor is electrically insulated from the rest of the device. The ERF case and gear box case stay aligned along the central axis by a large plastic bearing. The ERF housing is free to rotate about its axis and the force sensor measures a direct reaction force from the ERF housing.

## Results

A prototype of MR_CHIROD was built exactly as described in the previous section and was fully functional (Fig. 3). A detailed description of the MR_CHIROD components can be found in Table 2.

**Table 2:** MR_CHIROD's Component Details

**Name**	**Material**	**Description**
ERF Resistive Element Electrodes	Aluminum	Supply the necessary electric field to activate the ER fluid
ERF Resistive Element Case and Lid	Nylon	Housing of the ERF and electrodes
Power Trans. Shaft	Aluminum	Output shaft of ERF resistive element; Coupled to gear system; Optical Encoder shaft
Handles	Garolite	Haptic interface for the patient
Gear System	Brass	Multiplies the ERF resistive torque
Bearing	Plastic	Aligns the ERF case and gearbox
Plain Bearing	Brass	Aligns the electrodes; An electrical contact for the rotating electrode
Transmission Bracket	Aluminum	Rotates with ERF housing; Transmits hand force to gearbox
Seals	Teflon^®^	Prevents leakage
Screws, nuts, and washers	Plastic and Brass,	Fastener
Optical Encoder	Plastic	Renco Low-Profile Encoder with 1024/revolution resolution; Measures position, which is used to calculate velocity and acceleration
Force Sensors	Aluminum	FUTEK Load Cells, 10 lb Measures resultant torque of the ERF resistive element

The smart gripper exercise hand device is operated by closing it by compressing (squeezing) its handles, using four fingers on the top handle and the thumb on the bottom handle of the device, and is remotely adjusted to different levels of resistive forces. The device can also resist hand opening and finger extension. The angle of the gripper and the grip strength are monitored and recorded while the test is being performed. By knowing the position of the handle and reaction force of the ERF housing at small time steps, the dynamic performance can be captured and analyzed. The entire control process is accomplished in milliseconds by a computer where information can be gathered in fine detail. All of this data is saved on the computer so that insights can be gathered as to what is happening with the user.

### Test of MR_CHIROD outside of the MR environment

**Figure 4:**
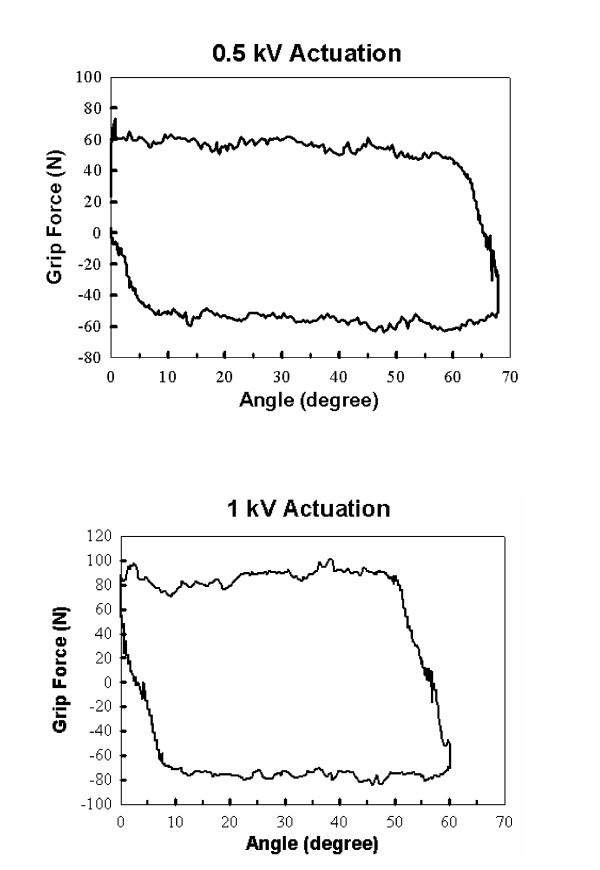
MR_CHIROD's force vs. angle diagram for 0.5 kV and 1 kV activation.

**Figure 5:**
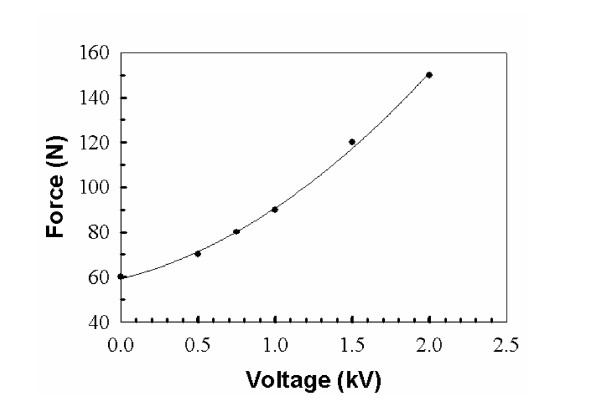
MR_CHIROD's force vs. voltage diagram.

Tests were performed outside of the MR scanner to verify the capabilities of the smart hand device. The force (grip strength) to open and close the gripper in various angle positions of the gripper for different voltage actuation was obtained. By applying the voltage through the ERF resistive element, the resistance of the ERF element is increased and more grip strength is needed to perform the exercise with the gripper. Fig. 4 shows the force versus angle when the ERF resistive element is activated with 0.5 and 1 kV. An average force output of the resistive element at each voltage (up to 2 kV) is plotted in Fig. 5. The results therefore suggest that the proposed system is capable of the desired force up to 150 N, depending on the level of applied voltage to the ERF resistive element.

### MR compatibility tests of MR_CHIROD

The following experiments were performed to demonstrate the compatibility of the ERF driven hand device using a 3-T Siemens Allegra 36 cm (gradient coil ID) head-only MRI, of the Athinoula A. Martinos Center for Biomedical Imaging located at the Massachusetts General Hospital of Boston, MA. Initial experiments were performed on each individual component of the device, and then the complete hand device was tested (Fig 3).

The power supply that supplied a voltage to the MR_CHIROD and the computer that reads the sensor data were located outside of the MRI room. A coaxial cable was used to connect the power supply to the test device. ERFs are magnet free in principle and it is shown in this section that they do not respond to any actuation by a magnetic field and that they are unaffected by the very strong field in the MR environment. To minimize Electromagnetic Interface (EMI), the wires were properly shielded and cables of appropriate size and impedance were used. The low amperage current required to activate the ERF ensures that the electromagnetic interference is kept to a minimum, both in the cables and the ERF components. The low amperage current also results in a low power consumption in the cables and ERF components, which avoids increased temperature in the test device.

Tests were performed to demonstrate that the magnetic field of the scanner does not apply forces on the MR_CHIROD, and using the device is safe in the MR environment. To evaluate the effects of the strong magnetic field on the function and performance of the components of the ERF driven hand device in the MR environment, the performance of the ERF, encoder, and force sensor was evaluated individually in the magnet. In addition, thorough testing was performed to study possible degradation of the MR images during the operation of the MR_CHIROD. Three different types of images were collected: a) Sagittal Tl-Weighted Localizer, b) Tl EPI images, c) Gradient echo EPI images. The following sub-sections provide details from all these tests.

#### Effect of magnetic forces on MR_CHIROD

To evaluate the effect of the magnetic forces, the ERF resistive element was placed inside a sealed plastic box with its outline scribed on a piece of paper below it. The box and ERF resistive element were advanced to the center of the magnet and back. The ERF resistive element lied within the pre-scribed outline, which means that it didn't move due to the magnetic forces from the scanner. Then the same test was repeated for the handles, the gear box, the optical encoder, and the force sensor. After all individual parts passed the test, the same test was performed on the complete hand device. The MR_CHIROD also lied within the outline and passed the test.

#### Performance evaluation of the ERF in MR environment

**Figure 6:**
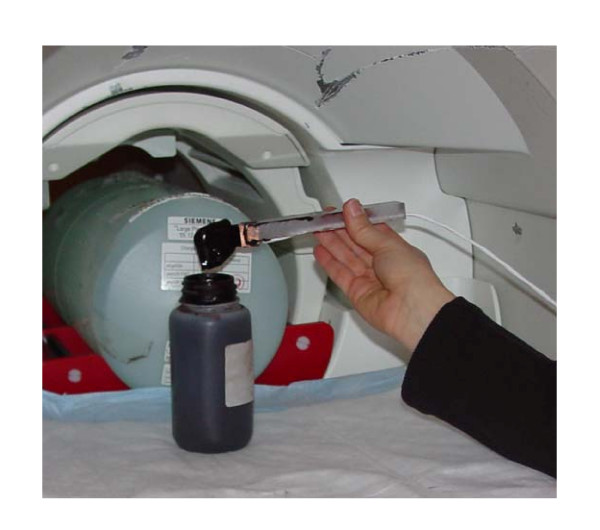
Simple device that was used to test the MR compatibility of ERFs.

**Figure 7:**
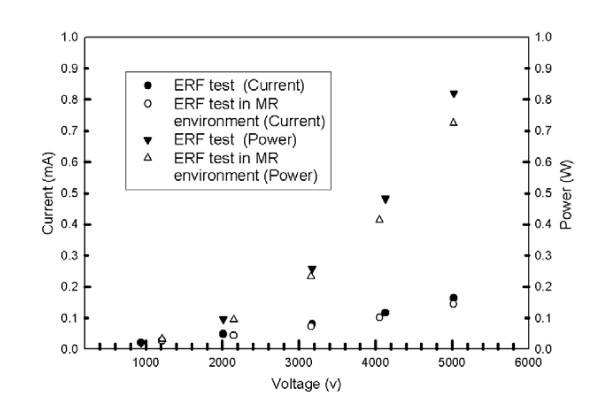
Current and power versus voltage for activated ERF.

To evaluate the effects of the strong magnetic field on the ERF in the MR environment, a simple device consisting primarily of two electrodes that were used to activate the fluid in between them was placed in the scanner (~400 mm from the isocenter of the magnet) shown in Fig. 6 and MR images were acquired. Visually, we verified that the activated ERF maintained the same properties and behaviour as when outside the MR scanner. Then the ERF was activated with different voltages (up to 5 kV) and the currents and voltages were recorded. Fig. 7 shows the plot of the current versus the voltage for the activated ERF inside the MR scanner (closed symbols) and outside the MR scanner (open symbols). It can be seen that the performance of the ERF has not been affected by the MR environment since upper (power) and lower (current) curves almost overlap. Then the ERF resistive element of the MR_CHIROD was placed in the same Gauss line and similar performance was observed with and without activation of the ERF.

#### Performance evaluation of the encoder in MR environment

**Figure 8:**
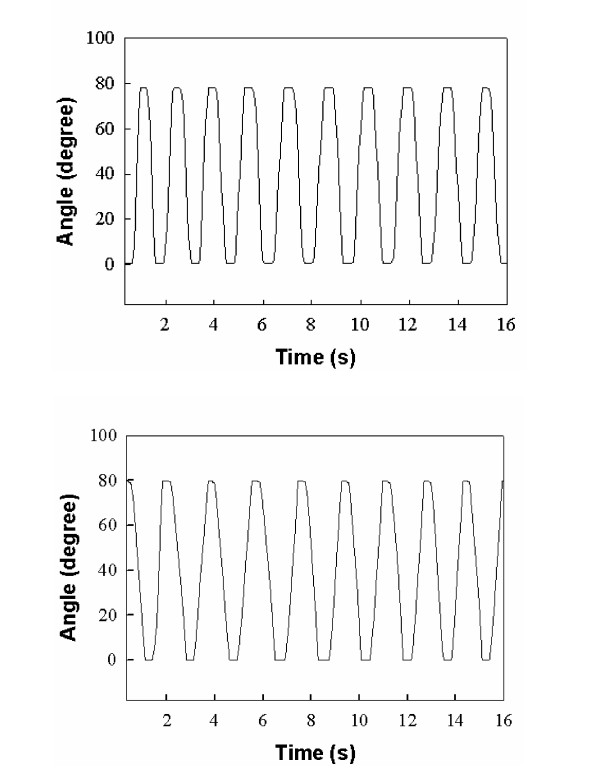
**Optical encoder MR compatibility**. outside the MR scanner (top); inside the MR scanner (bottom).

The optical encoder was attached to the input side of the gearbox and the handles were placed in the scanner (~400 mm from the isocenter of the magnet) and MR images were acquired to evaluate its performance in the MR environment. Then the handles were opened and closed and the encoder's direct reading of the handles' angles in real time were recorded in the computer. Fig. 8 shows the plot of the angle versus time when the device was outside the MR environment and when it was in zone 2. The results represent that the optical encoder works with no problem in the MR environment. It needs to be noted that in the graphs of Fig. 8 the frequency of opening and closing of the handles is slightly different as the two graphs correspond to two different tests performed by a human in two different instances and hence it is virtually impossible to repeat the exact same frequency.

#### Performance evaluation of the force sensor in MR environment

**Figure 9:**
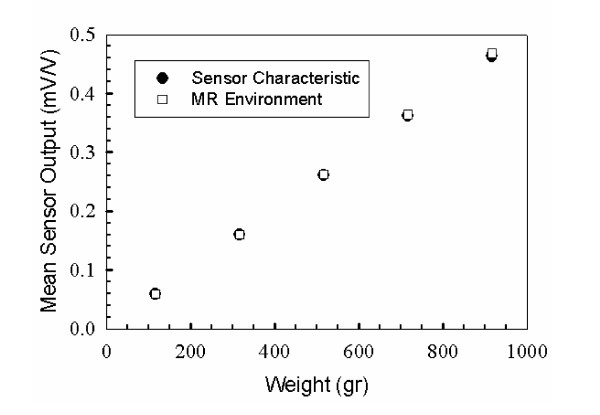
Force sensor MR compatibility.

To evaluate the performance of the force sensor in the MR environment, known weights were placed in the scanner (~400 mm from the isocenter of the magnet) and MR images were acquired. The electrical output of the sensor in mV/V was read by a computer, filtered and then converted to the corresponding force applied on the sensor using the output-load calibration equation provided by the manufacturer. A series of tests were performed for different weights. For a set of known weights, the signal output of the force sensor was obtained from the manufacturer's calibration equation and was plotted in Fig. 9 (black squares). The mean value of the sensor output for the same weights was obtained from MR tests and plotted in the same graph (Fig. 9 -white squares). The results of Fig. 8 show that the force sensor has the same accuracy inside and outside the MR environment. Similar tests were performed in zones 3 and 4 and all showed the same performance.

#### Effects of the operation of MR_CHIROD on MR images

**Figure 10:**
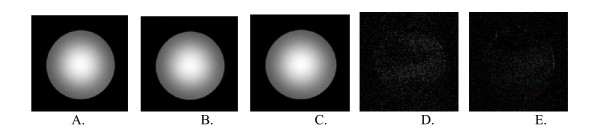
**Representative images acquired with the phantom**. **A**. Control, no device in MR scanner; **B**. ERF driven hand device in MR scanner (no ERF activation); **C**. ERF driven hand device in MR scanner and activated at 2 kV; **D**. Subtraction of A from B; **E**. Subtraction of A from C.

To ensure that using the ERF driven hand device had no degradation in the MR images and to visualize the possible artifacts caused by the introduction of the device in the magnetic field, a series of tests were performed. Each individual part of the hand device was placed in the scanner (~400 mm from the isocenter of the magnet) and the MR images were acquired. Then the complete hand device was placed in the scanner, and the images were acquired without the ERF activated, and with the ERF activated to see the possible effect that appears in the image by changing the voltage applied on the ERF. Various voltages up to 4 kV were used. We have to note here that the patient's hand will be located at a distance about 1 meter from the isocenter of the magnet or about 200 mT line when performing the rehabilitation exercises. The control image was acquired without any device. Three different types of images were collected: a) Sagittal Tl-Weighted Localizer (TR/TE = 2530/3.39 ms; Flip Angle = 7 degrees); b) Tl EPI images (TR/TE = 8000/30 ms, TE = 30 ms); and c) Gradient echo EPI images (TR/TE = 2000/30 ms, Flip Angle = 90 degrees). The imaging object was a cylindrical phantom filled with a solution of 1.24 g NiSO4 × 6 H2O/2.62 NaCl per 1000 g H2O. The phantom was placed in the head coil and driven into the magnet. As the phantom was kept immobilized, the images should stay identical. The obtained images looked identical for all experiments performed. Fig. 10 shows representative axial images when the complete hand device was inside 200 mT line experiments, and then the ERF was activated at 2 kV for slice [5/10]. Also, subtraction of the control image is listed. No image shift was found.

The signal to noise ratio (SNR) was calculated according to Firbank et al [34] in order to evaluate the noise and to see whether the introduction of ERF's in the magnetic field have affected the SNR of the MR images. The SNR values for axial images (slice [5/10]) during the 200 mT line experiments were calculated. For the single acquisition technique, four regions were drawn: a large circular region covering most of the test object, and three smaller circular regions placed on the background air pixel. The signal to noise ratio is given by: SNR = 0.655 × (S/SDair), where S is the mean signal intensity in the large circular region, and SDair is the average of standard deviation in the three smaller regions placed over air.

Table 3 shows the SNR values for each one of the device's individual components and the complete device assembly when they were placed inside the 200 mT line in the scanner. Results show that in all cases, the loss of SNR observed was not significant regardless of the ERF being activated in all zones where experiments were performed since 95% of the data points lie within ± 2 SD.

**Table 3:** Signal to Noise Ratio (SNR) of Images (device in 200 mT line)

**Description of the Test**	**Mean of SNR ± SD**
Control (no device)	145.46 ± 1.02
ERF Resistive Element	144.66 ± 1.48
Handles	145.43 ± 1.33
Handles and ERF Resistive Element	144.70 ± 0.93
Handles and Encoder	145.33 ± 0.74
Force sensor	144.90 ± 0.85
ERF hand device	144.65 ± 0.78
ERF hand device, actuated at 0.8 kV	144.53 ± 0.81
ERF hand device, actuated at 2 kV	144.40 ± 1.12

## Conclusion

Our novel force-feedback device designed for hand rehabilitation combines generation and measurement of high computer controlled resistive forces with a compact geometry and MR compatible structure. These properties were achieved by utilizing ERFs, which can produce large resistive forces upon activation with an electric field. It was demonstrated that ERF is MR compatible.

Tests with the first prototype showed that it was able to provide 160 N resistive forces for activation voltages up to 2 kV, which is approximately 50% of the maximum level of gripping force that a human hand can apply. Furthermore, our results demonstrated that the MR environment does not affect the ERF properties, the optical encoder, and the force sensor. The single acquisition technique showed that the ERF driven hand device had no degradation effect in the MR images.

We need to note that although the MR_CHIROD was designed and fabricated of nonmagnetic materials, the effect of Eddy currents on it was observed due to the presence of conductive electrodes. In the next prototype of the MR_CHIROD, the geometry of the electrodes will be changed so that continuous surfaces will be avoided. This will be achieved by introducing discontinuities with notches in the electrode surface or by using many smaller electrodes embedded in a non conductive material with the shape of the original electrode.

Current and future work includes: a) the development of an improved prototype, b) the development of closed loop torque control, and c) the performance of human tests outside and inside the MR environment. The main improvements that we will achieve in the second generation prototype will be the reduction of the frictional forces that were present in the first prototype due to the sealing used in the ERF resistive element and the reduction of the effect of Eddy currents.

## References

[B1] Rowe JB, Frackowiak RS (1999). The impact of brain imaging technology on our understanding of motor function and dysfunction. Curr Opin Neurobiol.

[B2] Hogan N, Krebs HI (1998). System and method for medical imaging utilizing a robotic device, and robotic device for use in medical imaging. US Patent 5,794,621.

[B3] Chinzei K, Kikinis R, Jolesz FA (1999). MR compatibility of mechatronic devices: design criteria. MICCAI '99 Lecture Notes in Computer Science.

[B4] Masamune K, Kobayashi E, Masutani Y, Suzuki M, Dohi T, Iseki H, Takakura K (1995). Development of an MRI-compatible needle insertion manipulator for stereotactic neurosurgery. J Image Guid Surg.

[B5] Felden A, Vagner J, Hinz A, Fischer H, Pfleiderer SO, Reichenbach JR, Kaiser WA (2002). ROBITOM-robot for biopsy and therapy of the mamma. Biomed Tech (Berl).

[B6] Larson BT, Erdman AG, Tsekos NV, Yacoub E, Tsekos PV, Koutlas IG (2004). Design of an MRI-compatible robotic stereotactic device for minimally invasive interventions in the breast. J Biomech Eng.

[B7] Chinzei K, Miller K (2001). Towards MRI guided surgical manipulator. Med Sci Monit.

[B8] Krieger A, Susil RC, Menard C, Coleman JA, Fichtinger G, Atalar E, Whitcomb LL (2005). Design of a novel MRI compatible manipulator for image guided prostate interventions. IEEE Trans Biomed Eng.

[B9] Ganesh G, Gassert R, Burdet E, Bleuler H (2004). Dynamics and control of an MRI compatible master-slave system with hydrostatic transmission. IEEE International Conference on Robotics and Automation.

[B10] Gassert R, Moser R, Burdet E, Bleuler H (2006). MRI/fMRI-compatible robotic system with force feedback for interaction with human motion. IEEE/ASME Transactions on Mechatronics.

[B11] Ku J, Mraz R, Baker N, Zakzanis KK, Lee JH, Kim IY, Kim SI, Graham SJ (2003). A data glove with tactile feedback for FMRI of virtual reality experiments. Cyberpsychol Behav.

[B12] Mraz R, Hong J, Quintin G, Staines WR, McIlroy WE, Zakzanis KK, Graham SJ (2003). A platform for combining virtual reality experiments with functional magnetic resonance imaging. Cyberpsychol Behav.

[B13] Cheng CP, Schwandt DF, Topp EL, Anderson JH, Herfkens RJ, Taylor CA (2003). Dynamic exercise imaging with an MR-compatible stationary cycle within the general electric open magnet. Magn Reson Med.

[B14] Diedrichsen J, Hashambhoy Y, Rane T, Shadmehr R (2005). Neural correlates of reach errors. J Neurosci.

[B15] Riener R, Villgrattner T, Kleiser R, Nef T, Kollias S (2005). fMRI-Compatible Electromagnetic Haptic Interface. IEEE-EMBS International Conference of the Engineering in Medicine and Biology Society.

[B16] Liu JZ, Dai TH, Elster TH, Sahgal V, Brown RW, Yue GH (2000). Simultaneous measurement of human joint force, surface electromyograms, and functional MRI-measured brain activation. J Neurosci Methods.

[B17] Hidler J, Mbwana J, Zeffiro T (2005). MRI compatible force sensing system for real-time monitoring of wrist moments during fMRI testing. IEEE International Conference on Rehabilitation Robotics.

[B18] Winslow WM (1949). Induced fibrillation of suspensions. J App Phys.

[B19] Kenaley GL, Cutkosky MR (1989). Electrorheological fluid-based robotic fingers with tactile sensing. IEEE International Conference on Robotics and Automation.

[B20] Wood D, Editorial (1988). Tactile displays: present and future. Displays- Technology and Applications.

[B21] Monkman GJ (1992). An Electrorheological tactile display.

[B22] Taylor PM, Hosseini-Sianaki A, Varley CJ (1996). Surface feedback for virtual environment systems using electrorheological fluids. J Modern Physics B.

[B23] Sakaguchi M, Furusho J (1998). Force display system using particle-type electrorheological fluids. IEEE International Conference on Rehabilitation Robotics.

[B24] Boese H, Berkemeier H-J (2000). Haptic device working with an electrorheological fluid. J Intelligent Material Systems and Structures.

[B25] Mavroidis C, Pfeiffer C, Celestino J, Bar-Cohen Y (2000). Controlled compliance haptic interface using electro-rheological fluids. Proceedings of SPIE – The International Society for Optical Engineering.

[B26] Mavroidis C, Bar-Cohen Y, Bouzit M, Bar-Cohen Y (2001). Chapter 19: Haptic interfaces using electrorheological fluids. Electroactive Polymer (EAP) Actuators as Artificial Muscles: Reality, Potentials and Challenges.

[B27] Weinberg B, Nikitczuk J, Fisch A, Mavroidis C (2005). Development of electro rheological fluidic resistive actuators for haptic vehicular instrument controls. Smart Materials and Structures.

[B28] Vitrani MA, Nikitczuk J, Morel G, Mavroidis C, Weinberg B (2006). Torque control of electrorheological fluidic resistive actuators for haptic vehicular instrument controls. J of Dynamic Systems, Measurement and Control, Transactions of the ASME.

[B29] Sakaguchi M, Furusho J, Genda E (1999). Basic study on rehabilitation training system using ER actuators. IEEE International Conference on Systems, Man, and Cybernetics.

[B30] Koyanagi K, Furusho J, Ryu U, Inoue A (2003). Development of rehabilitation system for the upper limbs in a NEDO project. IEEE International Conference on Robotics and Automation.

[B31] Kikuchi T, Furusho J, Oda K (2003). Development of isokineticexercise machine using ER brake. IEEE International Conference on Robotics and Automation.

[B32] Nikitczuk J, Weinberg B, Mavroidis C (2003). RehAbilitative Knee Orthosis Driven by Electro-Rheological Fluid Based Actuators. IEEE International Conference on Robotics and Automation.

[B33] Burdea G (1996). Force and Touch Feedback for Virtual Reality.

[B34] Firbank MJ, Coulthard A, Harrison RM, Williams ED (1999). A comparison of two methods for measuring the signal to noise ratio on MR images. Phys Med Biol.

